# Optimal tumor sampling for immunostaining of biomarkers in breast carcinoma

**DOI:** 10.1186/bcr2882

**Published:** 2011-05-18

**Authors:** Juliana Tolles, Yalai Bai, Maria Baquero, Lyndsay N Harris, David L Rimm, Annette M Molinaro

**Affiliations:** 1 Division of Biostatistics, Yale University School of Public Health, 60 College Street, New Haven, CT, 06511, USA; 2 Department of Pathology, Yale University School of Medicine, 333 Cedar Street, New Haven, CT, 06511, USA; 3 Department of Medical Oncology, Yale University School of Medicine, 333 Cedar Street, New Haven, CT, 06511, USA

## Abstract

**Introduction:**

Biomarkers, such as Estrogen Receptor, are used to determine therapy and prognosis in breast carcinoma. Immunostaining assays of biomarker expression have a high rate of inaccuracy; for example, estimates are as high as 20% for Estrogen Receptor. Biomarkers have been shown to be heterogeneously expressed in breast tumors and this heterogeneity may contribute to the inaccuracy of immunostaining assays. Currently, no evidence-based standards exist for the amount of tumor that must be sampled in order to correct for biomarker heterogeneity. The aim of this study was to determine the optimal number of 20X fields that are necessary to estimate a representative measurement of expression in a whole tissue section for selected biomarkers: ER, HER-2, AKT, ERK, S6K1, GAPDH, Cytokeratin, and MAP-Tau.

**Methods:**

Two collections of whole tissue sections of breast carcinoma were immunostained for biomarkers. Expression was quantified using the Automated Quantitative Analysis (AQUA) method of quantitative immunofluorescence. Simulated sampling of various numbers of fields (ranging from one to thirty five) was performed for each marker. The optimal number was selected for each marker via resampling techniques and minimization of prediction error over an independent test set.

**Results:**

The optimal number of 20X fields varied by biomarker, ranging between three to fourteen fields. More heterogeneous markers, such as MAP-Tau protein, required a larger sample of 20X fields to produce representative measurement.

**Conclusions:**

The optimal number of 20X fields that must be sampled to produce a representative measurement of biomarker expression varies by marker with more heterogeneous markers requiring a larger number. The clinical implication of these findings is that breast biopsies consisting of a small number of fields may be inadequate to represent whole tumor biomarker expression for many markers. Additionally, for biomarkers newly introduced into clinical use, especially if therapeutic response is dictated by level of expression, the optimal size of tissue sample must be determined on a marker-by-marker basis.

## Introduction

**Figure 1:**
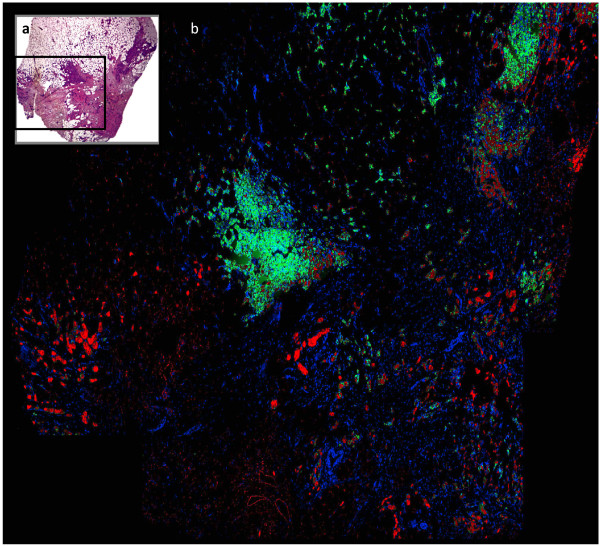
**Heterogeneity of MAP-Tau expression in a whole tissue section of breast carcinoma**. **(a) **H&E stain. **(b) **Immunofluorescence. Nuclei are labeled with DAPI. Cytokeratin is labeled with Cy3. MAP-Tau is labeled with Cy5.

Biomarkers have become essential for therapeutic decision-making and prognostication in breast carcinoma. Testing for Estrogen Receptor (ER), Progesterone Receptor (PR), and HER-2 is the standard of care; many other markers are also widely used [1]. However, conventional assays for biomarkers suffer from lack of objective methods of measurement. The most recent ASCO/CAP review of immunohistochemical assays for breast carcinoma found that 'up to 20% of ER and PR determinations worldwide may be inaccurate' [2]. The ASCO/CAP committee hypothesized that most misclassifications of ER and PR status are due to 'pre-analytical variables,' which are variations in tissue processing prior to immunostaining. However, an additional likely cause of the high rate of assay inaccuracy is biomarker heterogeneity [3]. Biomarkers are known to be heterogeneously expressed in breast carcinoma. Several investigations have demonstrated statistically significant differences in Estrogen Receptor expression between samples from the same tumor [4-6]. In addition, PR, HER-2, p53, and MIB-1 have been shown to have statistically significant differences in intra-tumor expression [5-8]. The heterogeneity of MAP-Tau epitope can be visualized in immunostained whole tissue sections (Figure 1). In the case of heterogeneous biomarkers, insufficient tumor sampling may lead to misclassification of biomarker status and inappropriate treatment. The phenomenon of biomarker heterogeneity in breast carcinoma has been well described, but no evidence-based standards have been developed for the size of tissue sample necessary to correct for heterogeneity in assays of biomarker status. The most recent set of ASCO/CAP guidelines states, 'large, preferably multiple core biopsies of tumor are preferred for testing if they are representative of the tumor (grade and type) at resection' [2]. To our knowledge, no prior investigations point to a more precise standard for the minimum number of cores or sections of resection tissue required to account for biomarker heterogeneity.

In order to estimate the required number of fields for accurate biomarker status assessment, we conducted a study of eight biomarkers that represent varying degrees of heterogeneity: ER, HER-2, AKT, ERK, S6K1, GAPDH, Cytokeratin, and MAP-Tau. First, we quantified the degree of heterogeneity for each marker using mixed-effects modeling. We then simulated sampling different amounts of tumor in order to determine the optimal number of 20X fields required to give a measurement of biomarker expression representative of the entire tissue sample. We hypothesized that markers with greater heterogeneity would require a larger number of sampled fields to produce a representative measurement.

## Materials and methods

### Cohorts

For this pilot study, two convenience samples were used, one from the clinical trial TAX 307 and the other from the tissue archives of the Pathology Department of Yale University. The first collection of subjects was a cohort (*n *= 122) from TAX 307, a prospectively collected, independent phase III clinical trial comparing TAC versus FAC. Patients were enrolled between January 1, 1998 and December 31, 1999, with a total of 484 patients randomized to receive either 5-fluorouracil-doxorubicin-cyclophosphamide (FACs; 75/50/500 mg/m2) or docetaxel-doxorubicin-cyclophosphamide (TAC; 500/50/500 mg/m2) as first line chemotherapy for metastatic breast cancer. All patients provided clinical consent prior to enrollment. Specimens and associated clinical information were collected under the guidelines and approval of the Dana Farber Human Investigation Committee under protocol #8219 to L.H.

The second collection of subjects consisted of 14 tumor resection specimens from patients who underwent surgery at Yale University/New Haven Hospital between 2001 to 2005. Whole tissue sections of formalin-fixed, paraffin-embedded primary invasive breast cancer tumors were obtained from the archives of the Pathology Department of Yale University. All the patients were diagnosed with infiltrating ductal carcinoma of the breast. All cases were judged to be ER-positive by pathologist-based scoring systems. None received chemotherapy or radiation prior to resection. The study was approved by the institutional review board for Yale University.

### Antibodies and quantitative immunofluorescence

MAP-Tau immunostaining was performed on the TAX 307 clinical trial cohort, which consisted of 122 whole section slides. Five *μ*m tissue sections from formalin-fixed paraffin-embedded tumor blocks were mounted on aminosilane glass slides (plus slides) and heated. Slides were immunostained using MAP-Tau monoclonal antibody which recognizes all human MAP-Tau isoforms independent of phosphorylation status (1:750; mouse monoclonal, clone 2B2.100/T1029, US Biological, Swampscott, MA). Slides were divided into six individual batches, each including one Breast Cancer Cell Line Control TMA slide. TAX 307 slides were incubated for 24 hours at 60°C. Slides were deparaffinized by oven incubation at 60°C for 20 minutes, followed by two 20 minute incubations in xylene. After slides were washed twice in 100% ethanol, once in 70% ethanol, and rehydrated with tap water, antigen retrieval by pressure cooking was performed in 6.5 mM sodium citrate buffer (pH 6.0) for 10 minutes. Endogenous peroxidase activity was quenched in methanol and 3% hydrogen peroxide for 30 minutes followed by rinsing in tap water and placement in 1× trisethanolamine-buffered saline (TBS; pH 8.0). Non-specific binding was reduced using a 30 minute preincubation in 0.3% bovine serum albumin (BSA) in 0.1 M tris-buffered saline (TBS, pH = 8) with 0.05% Tween (TBS-T). Slides were prepared for 4°C overnight incubation (12 hours) by adding a cocktail of MAP-Tau primary antibody (1:750) plus a wide-spectrum rabbit anti-cow cytokeratin antibody (Z0622; DAKO, Carpinteria, CA) diluted 1:100 in BSA/1X TBS-T. Following overnight incubation, slides were washed twice in 1× TBS with 0.05% Tween for 10 minutes and once in 1× TBS. Secondary antibody was then applied for one hour at room temperature. Goat antirabbit Alexa 488 (Molecular Probes, Eugene OR) was diluted 1:100 in horseradish peroxidase-conjugated EnVision antimouse secondary antibody (DAKO). Following incubation with secondary antibodies, slides were washed twice (ten minutes, then five minutes) in 1×TBS-T and once (five minutes) in 1×TBS. Cyanine-5 (Cy5) directly conjugated to tyramide (FP1117, Perkin-Elmer, Boston MA), diluted 1:50 in amplification diluent (Perkin-Elmer) was used as the fluorescent chromogen for target detection and was added to all slides for ten minutes at room temperature. Two final washes (ten minutes, then five minutes) in 1× TBS-T and one five minute wash in 1× TBS were performed. Slides were stained for double-stranded DNA using Prolong Gold mounting medium with anti-fade reagent 4',6-diamidino-2-phenylindole ('DAPI', Molecular Probes, Eugene OR). Normal breast epithelium served as internal positive controls while omission of the primary antibody served as the negative control for each immunostaining event.

For all epitopes other than MAP-Tau, immunostaining was performed on sets of serial slides from the second collection of subjects (*n *= 14) and the following protocol was used. Whole tissue sections were incubated at 60°C for 20 minutes before being deparaffinized with xylene, rehydrated, endogenous peroxidase blocked, and antigen-retrieved by pressure cooking for 15 minutes in citrate buffer (pH = 6). Slides were pre-incubated with 0.3% bovine serum albumin in 0.1 mol/L TBS (pH = 8) for 30 minutes at room temperature. The procedure for ERK staining was a follows: slides were incubated with a cocktail of ERK1/2 antibody diluted at 1:1,000 (Mouse monoclonal, clone L34F12; Cell Signaling Technology, Danvers, MA) and a wide-spectrum rabbit anti-cow cytokeratin antibody (Z0622; Dako Corp, Carpinteria, CA), diluted 1:100 in bovine serum albumin/TBS overnight at 4°C. This was followed by a 1-hour incubation at room temperature with Alexa 546-conjugated goat anti-rabbit secondary antibody (A11010; Molecular Probes, Eugene, OR) diluted 1:100 in mouse EnVision reagent (K4001, Dako Corp, Carpinteria, CA). Cyanine 5 (Cy5) directly conjugated to tyramide (FP1117; Perkin-Elmer, Boston, MA) at a 1:50 dilution was used as the fluorescent chromogen for ERK detection. Prolong mounting medium (Prolong Gold, P36931; Molecular Probes, Eugene, OR) containing 4',6-diamidino-2-phenylindole was used to identify tissue nuclei. Immunostaining for all remaining epitopes was done in a similar manner with antibodies as follows outlined in Table 1.

**Table 1:** Antibodies, epitopes, sources, and dilutions

Protein	Species	Clone	Dilutions	Supplier
ER	Mouse mAb	1D5	1:50	Dako
HER-2	Rabbit pAb	A0485	1:2,000	Dako
AKT	Rabbit mAb	11E7	1:1,000	CST
ERK1/2	Mouse mAb	L34F12	1:1,000	CST
S6K1	Rabbit mAb	49D7	1:450	CST
GAPDH	Rabbit mAb	14C10	1:500	CST
Cytokeratin	Rabbit pAb	Z0622	1:100	Dako

### Image capture and analysis

The automated quantitative analysis (AQUA) method of immunofluorescence allows exact measurement of protein concentration within subcellular compartments, as described in detail elsewhere [9]. In brief, a series of high-resolution monochromatic images were captured by the PM-2000 microscope (HistoRx). For whole tissue sections, multiple regions of interest (ROIs) containing invasive tumor were circled on the AQUA system screen based on the low-resolution cytokeratin (cytoplasm) image of the immunohistochemically stained slide taken with the AQUA system. The selected ROIs were automatically overlaid with a grid by the image capturing program and each 20X field of view (FOV) was defined automatically. For each FOV, in-focus and out-of-focus images were obtained using the signal from the 4',6-diamidino-2-phenylindole, cytokeratin-Alexa 546 and target protein-Cy5 channel. Target protein antigenicity was measured using a channel with emission maxima above 620 nm, in order to minimize tissue autofluorescence. Tumor was distinguished from stromal and non-stromal elements by creating an epithelial tumor 'mask' from the cytokeratin signal. The binary mask - in which each pixel is either 'on' or 'off' - is created on the basis of an intensity threshold set by visual inspection of FOVs.

The AQUA score of the target protein in each subcellular compartment was calculated by dividing the target protein compartment pixel intensities by the area of the compartment within which they were measured. AQUA scores were normalized to the exposure time and bit depth at which the images were captured; thus, scores collected at different exposure times are directly comparable.

### Statistical methods

Statistical analysis consisted of three steps: normalization, mixed-effects modeling, and estimation of optimal sampling via cross validation. ER and Tau data from different AQUA analyses were normalized to the same scale. Mixed-effects modeling was performed in order to estimate the coefficient of intra-tumor variation for each epitope. Mixed-effects models entail a rigorous statistical method for quantifying variation between repeated measurements from the same individual. Lastly, cross-validation of linear models was used to estimate the optimal number of FOVs necessary to produce a score representative of the whole tissue section for each epitope.

#### Normalization

Similar to other methods for quantitative immunofluorescence, AQUA scores are subject to some variation between analyses performed at different times. Potential sources of variation, such as buffer lot and microscope bulb hours, are numerous and impossible to completely eliminate. We therefore normalized AQUA scores between analyses performed at different times.

All epitopes with the exception of MAP-Tau and ER were processed in a single AQUA run and therefore did not require normalization. MAP-Tau and ER were run with standardized index arrays, consisting of breast carcinoma tissue and cell lines. To normalize scores of the experimental subjects for MAP-Tau and ER, quantile normalization was first performed on the index arrays. Next, a smoothing spline was fit to describe the transformation between the original index array scores and quantile-normalized index array scores. Lastly, the spline transformation was applied to the subjects' scores in order to transform them to the scale of the run selected as the baseline run. This normalization method has been validated on several independent cohorts for different carcinomas (Tolles *et al*., in preparation).

#### Mixed-effects modeling

Mixed effects model were fit for each epitope of interest. The form of the model was:

where *y_ijk _*is the AQUA score of the *i*th subject, in the *j*th ROI, at the *k*th FOV. *β*_0 _is the intercept term and *ε *is the residual. The model assumes , , and . The assumptions of normality for the random effects were verified with quantile-quantile plots. For some epitopes, plots of residuals against fitted values demonstrated heteroscedasticity, with . In those cases, we adjusted the model assumptions to account for this dependence. The coefficient of variation was calculated as . The R Language and Environment for Statistical Computing and NLME package were used for all computations [10].

#### Sampling simulation: model selection and cross-validation

**Figure 2:**
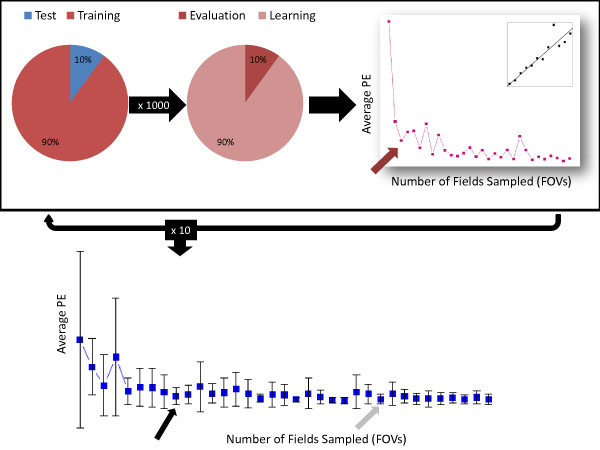
**Cross validation design**. (1) Division of cohort into test set and training set. Repeated 10 times. (2) Division of training set into learning set and evaluation set. Repeated 1,000 times. (3) Fitting of linear regression over learning set. Performed for sample sizes of one to thirty five field of views (FOVs). Calculation of average prediction error over evaluation set. Red arrow indicates first local minimum. (4) Calculation of average prediction error over the test set. Gray arrow indicates over local minimum over 10 training sets. Black arrow indicates smallest value within one standard error of average first local minimum.

Due to the inherent differences in the two cohorts, the analyses of the biomarkers differed slightly. However, in both, to choose the optimal number of fields (that is model selection) and estimate the corresponding prediction error we used two layers of resampling [11,12]. The first, or outer, layer was for estimating prediction error and the second, or inner, layer for model selection (see Figure 2).

For the MAP-Tau cohort, we employed 10-fold cross-validation for the first layer and Monte-Carlo cross-validation for the second. In the first layer the cohort was divided equally into ten groups. For each iteration, one of the groups served as an independent test set for calculation of prediction error while the other nine groups (that is 90% of the subjects) constituted the training set. In the second layer, this training set was subdivided into a learning set (90% of training set) and an evaluation set (10% of training set), for the purposes of selecting the optimal number of 20X FOVs. For each of the total 10 training sets, the learning and evaluation sets were both reconstituted 1,000 times. A linear regression model was fit to the subjects in the learning set. The corresponding independent variable was the average AQUA score of a subset of 20X FOVs sampled from each whole tissue slide, and the dependent variable was the overall average score for all FOVs on that slide. A separate regression was calculated for each potential number of FOVs (one to thirty five). Using the coefficients estimated from the regression model developed on the learning set, a predicted score was calculated for each subject in the evaluation set for every number of FOVs. The prediction error (PE) was calculated as follows for each number of FOVs and then averaged over the 1,000 evaluation sets:(1)

where *N*= # of subjects, , and *K *= # of fields in subject *i*. The first local minimum of the average prediction error was recorded.

Lastly, the mean PE for the independent test sets was calculated by averaging the PE over the 10 independent test sets for each potential number of FOVs (one to thirty five). The average first local minimum and standard error for the test set PE was recorded. In accordance with rules of parsimonious model selection [13], if there existed a model (here, a model is the number of FOVs) with mean PE within one standard error of that of the minimum model, the smaller model was selected as optimal. The entire process was repeated 100 times and the result averaged to produce a stabile estimate of the optimal number of FOVs. The standard deviation over the 100 repetitions was also calculated.

For all epitopes of interest other than MAP-Tau, the small number of FOVs measured for each subject required an alternative to the method of direct sampling used for MAP-Tau. Direct sampling would have introduced bias into the analysis, because of the relatively small number of FOVs available for each subject. For example, given a subject with only 10 FOVs, a sample of size of 10 would have consisted of all available FOVs from that subject's whole tissue section. Therefore, the average and standard deviation from each subject was used to describe a normal distribution. Then, randomly generated observations from that normal distribution were sampled as above.

For epitopes other than MAP-Tau, in the first layer, leave-one-out cross-validation was used in place of 10-fold cross-validation. That is, in each iteration of the cross-validation, the test set consisted of one subject and the remaining subjects constituted the training set. Again, in the second layer, the training set was subdivided into learning and evaluation sets. However due to the small sample sizes, instead of Monte-Carlo cross-validation, we employed bootstrap sampling, in which a training set of size *n *was sampled with replacement to create a learning set of size *n*. Subjects not selected for the learning set made up the evaluation set. A linear model was used in a similar manner as for MAP-Tau and an optimal number of FOVs was selected by averaging the prediction error in the evaluation set over 1,000 iterations of the training set splitting procedure. Test set error was calculated in the same manner as for MAP-Tau and the one-standard-error parsimony rule again applied to select the final 'optimal' number of FOVs. As in the MAP-Tau cohort, the entire process was repeated 100 times and the average and standard deviation calculated.

In order to test the validity the simulated sampling method used for these epitopes, an additional analysis was performed on the MAP-Tau data. For each of the 122 subjects, a subset of 20 FOVs was randomly sampled from all FOVs available. Randomly generated values from a normal distribution described by the mean and variance of the 20 FOV subset was then used for selection of optimal number of FOVs and calculation of prediction error was then performed.

For all epitopes, to assess how close the predicted value was to the overall average AQUA score, we computed the absolute distance of the two values divided by the standard deviation of AQUA scores for each person as:(2)

where *N*, , and *K *are defined in Equation 1 and . This value was then averaged over the layers of cross-validation resulting in an average absolute standardized score. The R Language and Environment for Statistical Computing was used for all computations.

## Results

### Mixed-effects analysis of intra-tumor heterogeneity

**Figure 3:**
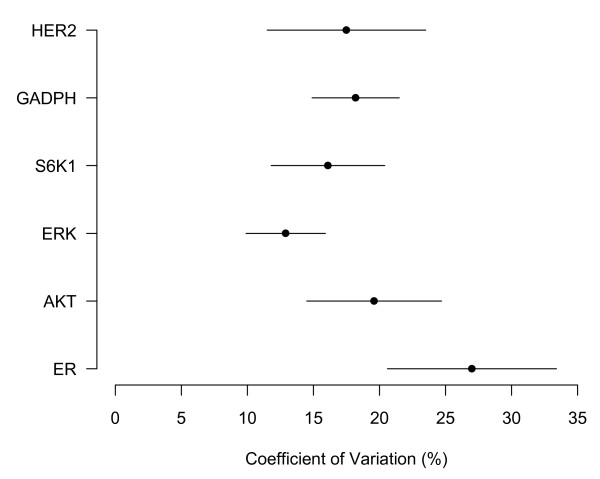
**Coefficient of variation (%) by epitope with 95% confidence intervals**.

We calculated an average intra-tumor coefficient of variation by epitope via a mixed-effects model fit to the AQUA scores from the 20X FOVs. Results appear in Figure 3 and are expressed as percentages with 95% confidence intervals. Overlapping intervals indicate that there is no significant difference between the coefficients of variation. Information about the location of FOVs in ROIs on the whole tissue slide was not collected for MAP-Tau and cytokeratin proteins; it therefore was not possible to calculate a coefficient of variation for these epitopes. The only significant difference is between the coefficients for ERK and ER. Of note, the 'housekeeping' protein GAPDH, which we expected to show relatively homogeneous expression, has a coefficient of variation that is not statistically significantly different from that of ER or HER-2. Only cases judged to be ER-positive by pathologist-based scoring systems were included in the analysis; models based on a heterogeneous population of ER-negative and ER-positive cases might have overestimated inter-tumor variation or underestimated intra-tumor variation for ER-positive cases.

### Cross-validated optimal number of FOVs

For each epitope of interest, we simulated taking one to thirty five FOVs for a subset of subjects (the learning set). We then used the average AQUA score of the sampled FOVs to develop a linear model. The model was used to the predict scores for a distinct group of subjects, the test set, from which the same number of FOVs were sampled. Next, we calculated the PE, which is the average squared error from each set of predictions over the test set. We repeated this simulation with different learning and test sets, as described in the methods. Lastly, we located the average first local minimum of the PE and recorded the smallest number of FOVs within one standard error of this minimum. The result appears in the first column of Table 2. Also shown are the standard error of the estimate and the corresponding average absolute standardized score (Equation 2).

**Table 2:** Optimal number of fields by epitope with prediction error

Marker	Optimal number of 20X field of views	SE of optimal number(field of views)	Average absolute standardized score (Equation 2)
ER	8	3.4	.31
HER-2	5	3.0	.56
AKT	4	1.5	.65
ERK	6	2.5	.31
S6K1	6	3.4	.21
GAPDH	12	4.1	.24
Cytokeratin	3	4.3	.41
MAP-Tau	14	4.2	.60
MAP-Tau (direct sampling)	14	4.2	.55

The optimal number of fields for epitopes ranged from three to fourteen. Standard error of the estimate ranging from 1.1 to 4.2, demonstrating that the estimates generated were stable. There are significant differences in the optimal number of FOVs between some of the epitopes. These differences roughly correlate with the results of the mixed-effects analysis of heterogeneity: the coefficients of variation for ER, HER-2, AKT, S6K1 were not found to be significantly different and, correspondingly, the optimal FOV results for these epitopes are similar. Cytokeratin and MAP-Tau, for which it was not possible to calculate coefficients of variation, have optimal numbers of FOVs of three and fourteen respectively. Given the qualitative heterogeneity of MAP-Tau on visual analysis and contrastingly ubiquitous expression of cytokeratin in breast carcinoma, these results support the hypothesis that markers with greater heterogeneity have a larger optimal number of FOVs. However, the correspondence between biomarker heterogeneity and optimal number of FOVs was not perfect: ER and ERK had significantly different coefficients of variation and yet had optimal number of FOVs of eight and six respectively. The average absolute standardized score at the optimal number of fields is reported as an average distance in terms of a subjects' AQUA score standard deviation. For example, for ER, a subject's predicted score, as calculated from the optimal number of FOVs, will, on average, differ from the subject's 'true' score by .31 standard deviations. The average absolute distance at the optimal number of FOVs varies slightly between epitopes but remains below one standard deviation for all but one epitope. Again, only ER-positive cases were analyzed in order to avoid bias in the estimate of the optimal number of FOVs for ER.

As described in the methods, due to the small sample size and number of FOVs, the biomarkers besides MAP-Tau were imputed by simulating from a normal distribution based on the observed mean and standard deviation of the each individual biomarkers. To test the validity of this imputation, we performed the simulation with MAP-Tau and the results were almost identical to the results when we employed direct sampling of observed data (Table 2).

## Discussion

We investigated biomarker heterogeneity and the optimal number of FOVs required for accurate immunostaining assessment of biomarker expression in breast carcinoma. Our mixed-effects analysis showed that, between the eight biomarkers we examined, there were significant differences in heterogeneity, as quantified by the intra-tumor coefficient of variation. Optimal number of 20X FOVs, determined by the cross-validated average prediction error, varied by epitope from three to fourteen. The clinical significance of our findings is two-fold. First, they suggest that biopsies consisting of very few FOVs may be inadequate for use in diagnostic immunostains, because they may not contain enough FOVs to account for biomarker heterogeneity. Second, they suggest that the optimal tissue sampling algorithm required to account for biomarker heterogeneity must be determined individually for each biomarker introduced into clinical use. The optimal number of FOVs trended with the results of the mixed-effects analysis of heterogeneity. S6K1, ERK, and AKT had similar optimal FOV sample sizes and a correspondingly large overlap in the 95% confidence intervals for their coefficients of variation. ER, which had the highest measured coefficient of heterogeneity, had a relatively large optimal sample size. Although it was not possible to calculate a coefficient of variation for MAP-Tau, its large optimal FOV sample size is consistent with the qualitative heterogeneity observed in immunostains. The similarity of the optimal number of FOVs between ER and ERK, despite significant differences in their coefficients of correlation, demonstrates imperfect correspondence between mixed-effects modeling of heterogeneity and the optimal number of FOVs. This suggests that optimal sampling must be empirically calculated for each marker rather than predicted from models of marker heterogeneity.

Of note, we included only ER-positive cases, as judged by pathologist-based scoring systems, in our analysis. We predicted that ER-negative cases would likely have an extremely low intra-tumor variability. Therefore, analysis of a mixed sample of ER-negative and ER-positive cases might have underestimated both intra-tumor variability and the optimal number of FOVs. We do not believe that this limits the generalizability of our results, as our goal was to estimate a minimum number of FOVs required for accurate determination of ER status.

The differences between the optimal number of FOVs for the biomarkers we tested suggests that there exists no single, optimal sampling algorithm for all biomarkers in breast carcinoma. Instead, the optimal number must be determined on a marker-by-marker basis. Biomarkers that are known to be more heterogeneous, such as MAP-Tau, are likely to require more FOVs; however, for the reasons stated above, precise sampling algorithms must be empirically determined.

The observed heterogeneity likely arises from several sources: intrinsic biological differences in epitope expression, pre-analytic variables (such as variable cold ischemic time and formalin penetration of tissue), and technical variables of the AQUA method of quantitative immunofluorescence. As it is impossible to know *a priori *the relative contributions of the different sources variability, we believe that blind adjustment of the assay to reduce its dynamic range risks the loss of clinically relevant information. Instead, we believe that the best strategy is to first determine the degree of sampling necessary to produce a representative score and then to compare that score to cutoffs that have been validated against clinical outcomes.

This study has several limitations. First, we used the average AQUA score over all FOVs in a whole tissue slide to model the 'true' representative score for each subject when calculating prediction error. The variation within a single whole tissue slide may be less than the variation between histologic 'blocks' from different regions of tumor. As a result, the number of FOVs determined in this study may underestimate the amount required to obtain a representative measure for each biomarker's expression. Our results may be conservatively interpreted as a minimum required number for clinical use.

A second limitation is the relatively small number of subjects used for many of the biomarkers. For all biomarkers other than MAP-Tau, we were required to simulate sampling FOVs from a normal distribution described by the measured mean and variation of observed FOVs, in order to avoid introducing bias. However, the validity of this analysis of the smaller cohort (*n *= 14) is strongly supported by our dual analysis of MAP-Tau, which was a large cohort (*n *= 122) with a large number of FOVs measured per subject. When MAP-Tau data was analyzed by both direct sampling and simulation, the results for the optimal number of fields and SE of the estimate were identical.

The third limitation is that AQUA is not currently used in many clinical laboratories. AQUA uses fluorescence for visualization and optimal quantification rather than DAB used in most conventional labs. However, the underlying immunohistochemistry technique and biology is the same, so the results should be generalizable to any method of visualization. Furthermore, the most recent set of ASCO/CAP guidelines states, 'image analysis is a desirable method of quantifying percentage of tumor cells that are immunoreactive' [2].

This pilot study offers guidance regarding the size of tissue sample that is required to account for heterogeneity in the specific biomarkers studied. More broadly, it suggests that further investigations are necessary in order to describe optimal sampling for other biomarkers in pre-clinical or clinical use, both in breast carcinoma and other tissue types.

## Conclusions

Our results demonstrate that appropriate tumor sampling to account for biomarker heterogeneity varies by marker and should be determined on an individual basis for all new markers considered for clinical use. Furthermore, our results suggest that, for some markers, core biopsies with only a few fields of tumor may represent inadequate samples. The implication for clinical practice is that number of fields assessed is a critical parameter for companion diagnostic tests and should be optimized prior to introduction of new biomarker assays.

## Abbreviations

ASCO/CAP: American society of clinical oncology/college of American pathologists; AQUA: automated quantitative analysis; BSA: bovine serum albumin; DAB: 3,3'-diaminobenzidine; DAPI: 4',6-diamidino-2-phenylindole; ER: estrogen receptor; ERK: extracellular signal-related kinase; FAC: 5-fluorouracil-doxorubicin-cyclophosphamide; FOV: field of view; GAPDH: glyceraldehyde 3-phosphate dehydrogenase; PR: progesterone receptor; ROI: region of interest; TAC: docetaxel-doxorubicin-cyclophosphamide; TBS: trisethanolamine-buffered saline; TMA: tissue microarray.

## Competing interests

Dr. Rimm is a founder, stockholder, and consultant to HistoRx, Inc.

## Authors' contributions

JT performed the statistical analyses and drafted the manuscript. MB and YB carried out the AQUA assays. LNH was responsible for tissue acquisition from TAX 307 cohort. DLM conceived of the study, participated in its design, and prepared the manuscript. AMM designed the statistical analyses and prepared the manuscript. All authors read and approved of the final manuscript.
